# Golgin-160 and GMAP210 play an important role in U251 cells migration and invasion initiated by GDNF

**DOI:** 10.1371/journal.pone.0211501

**Published:** 2019-01-29

**Authors:** Chuan-Xi Tang, Lan Luan, Lin Zhang, Yue Wang, Xin-Feng Liu, Jie Wang, Ye Xiong, Dan Wang, Lin-Yan Huang, Dian-Shuai Gao

**Affiliations:** 1 Department of Neurobiology and Anatomy, Xuzhou Key Laboratory of Neurobiology, Xuzhou Medical University, Xuzhou, Jiangsu, China; 2 School of Nursing, Xuzhou Medical University, Xuzhou, Jiangsu, China; 3 Department of Neurosurgery, The Affiliated Hospital of Xuzhou Medical University, Xuzhou, Jiangsu, China; 4 School of Medicine information, Xuzhou Medical University, Xuzhou, Jiangsu, China; 5 School of Medical Technology, Xuzhou Medical University, Xuzhou, Jiangsu, China; University of South Alabama Mitchell Cancer Institute, UNITED STATES

## Abstract

Gliomas are the most common malignant tumors of the brain and are characteristic of severe migration and invasion. Glial cell line-derived neurotrophic factor (GDNF) promotes glioma development process. However, the regulatory mechanisms of promoting occurrence and development of glioma have not yet been clearly elucidated. In the present study, the mechanism by which GDNF promotes glioma cell migration and invasion through regulating the dispersion and location of the Golgi apparatus (GA) is described. Following GDNF treatment, a change in the volume and position of GA was observed. The stack area of the GA was enlarged and it was more concentrated near the nucleus. Golgin-160 and Golgi microtubule-associated protein 210 (GMAP210) were identified as target molecules regulating GA positioning. In the absence of either golgin-160 or GMAP210 using lentivirus, the migration and invasion of U251 cells were decreased, while it was increased following GDNF. It was also found that the GA was decreased in size and dispersed following golgin-160 or GMAP210 knockdown, as determined by GA green fluorescence assay. Once GDNF was added, the above phenomenon would be twisted, and the concentrated location and volume of the GA was restored. In combination, the present data suggested that the regulation of the position and size of the GA by golgin-160 and GMAP210 play an important role in U251 cell migration and invasion.

## Introduction

Glioma is a heterogeneous, highly complicated central nervous system (CNS) tumor with an uncertain mechanism of initiation and progression[1], which results in an unfavorable outcome. The invasion properties of glioblastoma render a radical surgery necessary and are responsible for its recurrence[2]. In addition, the migration and invasion of glioma cells severely disrupt brain function, due to the disruption of normal astrocytes, which are lifted up from blood vessels by glioma cells[3, 4]. So, it remains a holy grail of the migration of glioma cells.

Cell migration is crucial for remodeling and regulating brain function [5], both during the early development phase[6] and adulthood. What is then the difference between a normal and a pathological brain? In normal adult brains, cell migration is limited and appears mainly in the sub ventricular zone and dentate gyrus areas [5]. Stem cells located in these two areas continuously produce progenitors that migrate and differentiate. Cell migration is also a feature of malignant gliomas that use the same tortuous route traveled by stem cells[7]. Many molecules, including glial cell line-derived neurotrophic factor (GDNF), are involved in cell migration. GDNF contributes to the maintenance of neuronal migration toward the olfactory bulb [8]. In a previous study, Xiong *et al* reported that GDNF could activate the proN-cadherin mediated intracellular signal transduction in glioma cells, which promotes the secretion of matrix metalloproteinase-9 and degrades extracellular matrix[9]. It therefore appears that GDNF plays a role in promoting cell migration. Several studies have focused only on the cell migration and the associated signaling molecules activated by GDNF. Rather, little attention has been paid to the dynamic changes in the movement of the cells themselves.

Fibroblast polarization is one of the most important phenomena in directional cell migration[10]. In cell polarization, the Golgi apparatus (GA) is critically involved in directional cell migration, since GA acts a pivotal part in supplying the membrane components to the leading edge for membrane protrusion when the cell is moving[11, 12]. The asymmetric distribution of protrusional activity is a general characteristic of directional motility[13], which requires the integrity of GA and microtubules (MTs). Further, the reorientation of GA has an active role in directed secretion and cell polarity[14]. The ability of the GA to nucleate MTs has recently been demonstrated, and the molecular machinery involved in the position of GA has been partly identified. Studies have confirmed that various treatments that disrupt Golgi architecture are accompanied by an inhibition of cell migration. For example, deletion of golgin-160 or Golgi microtubule-associated protein 210 (GMAP210) led to fragmentation and disperse of the GA without disassembling microtubule or actin cytoskeletal systems, and contributed to the inhibition of directional cell migration [15, 16].

It has been identified GDNF promotes migration and invasion of glioma cells[9]. The changes in the morphology and position of GA were examined following treatment of GDNF. For this, we used lentivirus to disrupt the expression of golgin-160 or GMAP210, resulting in the inhibition of U251 cell migration and invasion with or without GDNF. This suggested that GA is a prerequisite for directed glioma cell migration with GDNF treatment. The aim of the present study was designed to figure out the unique biology of GDNF on the migration and invasion of glioma cells, which provides hitherto unrevealed the mechanism from the perspective of the GA.

## Material and methods

### Cell culture and GDNF treatment

Human U251 glioma cells [Obio Technology (Shanghai) Corp., Ltd, Shanghai, China] were cultured in a 37°C, 5% CO2 humidified incubator using Dulbecco’s modified Eagle’s medium (DMEM, SH30022.01, Hyclone, GE Healthcare Life Sciences, Logan, UT, USA) containing 10% fetal bovine serum (FBS, SV30087.02, GE Healthcare Life Sciences), 100U/ml penicillin and 100U/ml streptomycin. Exponentially growing cells, derived after two or three passages, were used for the following experiments: i) U251 cells treated with 50ng/ml GDNF (Merck KGaA, Darmstadt, Germany) for 0, 0.5, 24 or 48h were used to detect the effects of GDNF on the stack area of GA; ii) U251 cells treated with various concentrations of GDNF (0, 25, 50, 75ng/ml) for 48h were used to determine the optimal GDNF concentration.

### Golgi apparatus green fluorescence assay

U251 cells treated with GDNF were fixed with 4% formaldehyde for 20min at room temperature and permeabilized with 0.3% Triton X-100 for 20min. Then GOLGI ID Green assay kit[17] (ENZ-51028-K100, Enzo Life Sciences, Inc., Farmingdale, NY, USA) was used for aldehyde-fixed cells staining, in order to localize and quantify the GA. Golgi ID green allowed the Golgi apparatus to be identified in confocal images.

### Immunofluorescence & confocal microscopy

U251 cells treated with GDNF were fixed with 4% formaldehyde for 20min at room temperature and permeabilized with 0.3% Triton X-100 for 20min. Next, the cells were blocked with 5% goat serum in PBS for 20min, followed by immunostaining with the primary antibodies (rabbit anti-Golgin-160, ab96080, Abcam, 1:100; Abcam, Cambridge, UK) overnight at 4°C. After washing with PBS, the cells were incubated with secondary antibody (DyLight 594-conjugated goat anti-rabbit, E032420, 1:500, EarthOx Life Sciences, Millbrae, CA, USA) for 40min at 37°C in a dark, moist environment. DAPI was applied to stain the nuclei, and fluorescence images were captured with fluorescence or confocal laser fluorescence microscopes (FV10i, Olympus Corporation, Tokyo, Japan).

### Western blot

Total protein in each sample was extracted with NP-40 lysis buffer. The protein concentration in each sample was detected using the BCA protein assay kit (Hyclone-Pierce, Rockford, IL, USA). The protein samples were separated on an 8% SDS-PAGE gel and then transferred to PVDF membranes. After blocking by 5% skimmed milk for 1h at room temperature, blots were incubated with primary antibodies: rabbit anti-Golgin-160 (ab9608; 1:1000; Abcam), mouse anti-GMAP-210 (sc-135928; 1:500; Santa Cruz Biotechnology, Inc., Dallas, TX, USA), mouse anti-GAPDH (AG019; 1:10000; Beyotime Institute of Biotechnology, Haimen, China) and anti-actin (1:10000; Santa Cruz Biotechnology, Inc. sc-8432) antibodies at 4°C overnight. Next, blots were incubated with corresponding goat anti-rabbit (1:3000; Santa Cruz Biotechnology, Inc.) and goat anti-mouse (1:5000; Santa Cruz Biotechnology, Inc. sc-362257, sc-362277) secondary antibodies for 2h at room temperature in the dark. Finally, the membranes were scanned using Odyssey imaging system (LI-COR Biosciences, Lincoln, NE, USA) and quantified with ImageJ 1.48v (National Institutes of Health, Bethesda, MD, USA). GAPDH was used as the internal reference protein.

### Golgin-160 and GMAP-210 knockdown in U251 cells by lentiviral RNA

pLKD-CMV-R&PR-U6-shRNA vectors were generated using the RNAi sequences (human Golgin-160: 5-GGAGATGAAGACCAAACAT-3, GMAP210: 5-GCAGTTGACACAACTTATA-3; control: 5-TTCTCCGAACGTGTCACGT-3) and transfected into DH5α cells. The packaged lentivirus containing supernatant was collected and then used to infect U251 cells. Through screening with puromycin for one week, the knockdown of Golgin-160 (KD-Golgin-160) and GMAP210 (KD-GMAP-210), as well as their negative control cells (control), were obtained and stably expressed the Golgin-160-, GMAP210- and vector- specific RNAis (Control), respectively. The blank group was made up of normal U251 cells that did not undergo any treatment.

### Reverse transcription quantitative polymerase chain reaction (RT-qPCR)

Total RNA was extracted from U251 cells and its concentration and quality were measured using an ultraviolet spectrophotometer (OD-1000, BioDee Biotechnology Co. Ltd, Bejing, China). cDNA was synthesized through reverse transcription of RNA. Next, quantitative PCR was used to determine the mRNA expression of Golgin-160 and GMAP210. PCR conditions were as follows: i) 95°C for 30s; ii) 40 cycles: 95°C for 5s, 60°C for 34s; iii) 95°C for 1min, 55°C for 1min; iv) 81 cycles: 55.0°C-95.0°C, with every cycle increasing the setpoint temperature by 0.5°C and resting for 4s. Actin was used as the internal reference gene. The relative mRNA expression levels of the target genes were calculated using the 2^-ΔΔCt^ method. The primer sequences were as follows: Actin forward: 5-TTCTACAATGAGCTGCGTG-3 and reverse, 5-CTCAAACATGAT CTGGGTC-3; Glogin-160 forward: 5-GGAAACACACTTGCAGTCGT-3 and reverse, 5-TCTTC TGCTTCTGTTCCGTG-3; GMAP-210 forward: 5-GGAGGAGATGGAGCAGTTGT-3 and reverse, 5-CCACAGATTGCTGATTTGGTC-3.

### Cell proliferation assays

Cell proliferation was detected via the Cell Counting Kit-8 assay (CCK-8; Dojindo Laboratories, Kumamoto, Japan).The U251 cells were seeded into 96-well plates at a density of 5x10^3^ cells/ well. The first CCK8 assay was performed when cells treated with non-serum were starved for 12 h. The above-mentioned results were regarded as starting value (0 h). Meanwhile, different treatment conditions were carried out as follows: serum-free group, serum-free + DNA inhibitor +GDNF group, serum-free + DNA inhibitor group, serum-free + GDNF group, DMEM+ serum group. The concentration of GDNF was 50ng/ml. DNA inhibitor is mitomycin C (No.10107409001, Roche). 10μl CCK8 dye was added to the wells and incubated for 60min at 37˚C. The optical density was assessed at a wavelength of 450 nm with UVmax kinetic microplate reader (Molecular Devices, Wokingham, UK). Then we checked the CCK-8 results at the indicated time-points (0,12h and 48h) and recorded the 60min OD450 value.

### Wound healing assay

Cell migration was evaluated by wound healing assay. U251 cells were seeded in six-well plates (10^6^/well) and cultured until confluence. Following culture with serum-free DMEM for 12 h to avoid the effect of cell proliferation, cells were wounded manually using a 20-μl pipette tip. The remaining cells were rinsed with PBS and cultured with serum-free DMEM. Photographs were taken at a magnification of x100 at 0, 24 and 48 h to monitor cell migration across the wound. The decrease in wound area was measured using IPP software.

### Live cell imaging assay

Live cell motility imaging was captured using the Olympus IX81 inverted microscope with a new UIS2 optical system. U251 cells were seeded in six-well plates (10^6^/well) and cultured until 80% confluence. Following culture with serum-free DMEM for 12 h, cells were wounded manually using a 20-μl pipette tip. Meanwhile, GDNF was added and the concentration was 50ng/ml. Images were acquired every 20 mins in the phase starting from 6h to 48h.

### Transwell matrigel invasion assay

Serum-free DMEM (100μl and 700μl) were respectively put into the top and bottom chamber of 24-wel plates Transwell chamber for 2h at 37°C, and then removed. After the chamber was coated with Matrigel for 4h, cells (3×10^4^) cultured with serum-free DMEM for 12h were seeded into the top chamber and incubated with DMEM containing 10% FBS for 24h at 37°C. Cells were fixed with 4% formaldehyde and stained with crystal violet staining solution. The invading cells were photographed (magnification, ×200) and counted.

### Image analysis

After confocal images were acquired, the number of fluorescent objects per cell was determined using a fixed threshold and the “Analyze Particle” plugin in Image Pro Plus (IPP) software. The stack area and fluorescence intensity of Golgi objects were determined for each cell using IPP. The integrated optical density (IOD) was calculated by dividing the GA fluorescence intensity by the stack area. We defined the distance from the GA margin to the center of the nucleus by selecting the best fitting circle around the nucleus. Then the radius of the circle was measured at the same standard scale.

### Statistical analysis

All analysis was performed with the SPSS 19.0 (SPSS, Inc., Chicago, IL, USA) and expressed as the mean ± standard deviation (SD). An independent sample t-test was used to determine significant differences in the mean values between two groups. Multiple comparisons between groups were performed using one-way analysis of variance followed by the pairwise comparison (LSD) test for statistical analysis. P<0.05 was considered to indicate a statistically significant difference.

## Results

### 1. GDNF increased the stack area of the GA in U251 cells

**Fig 1:**
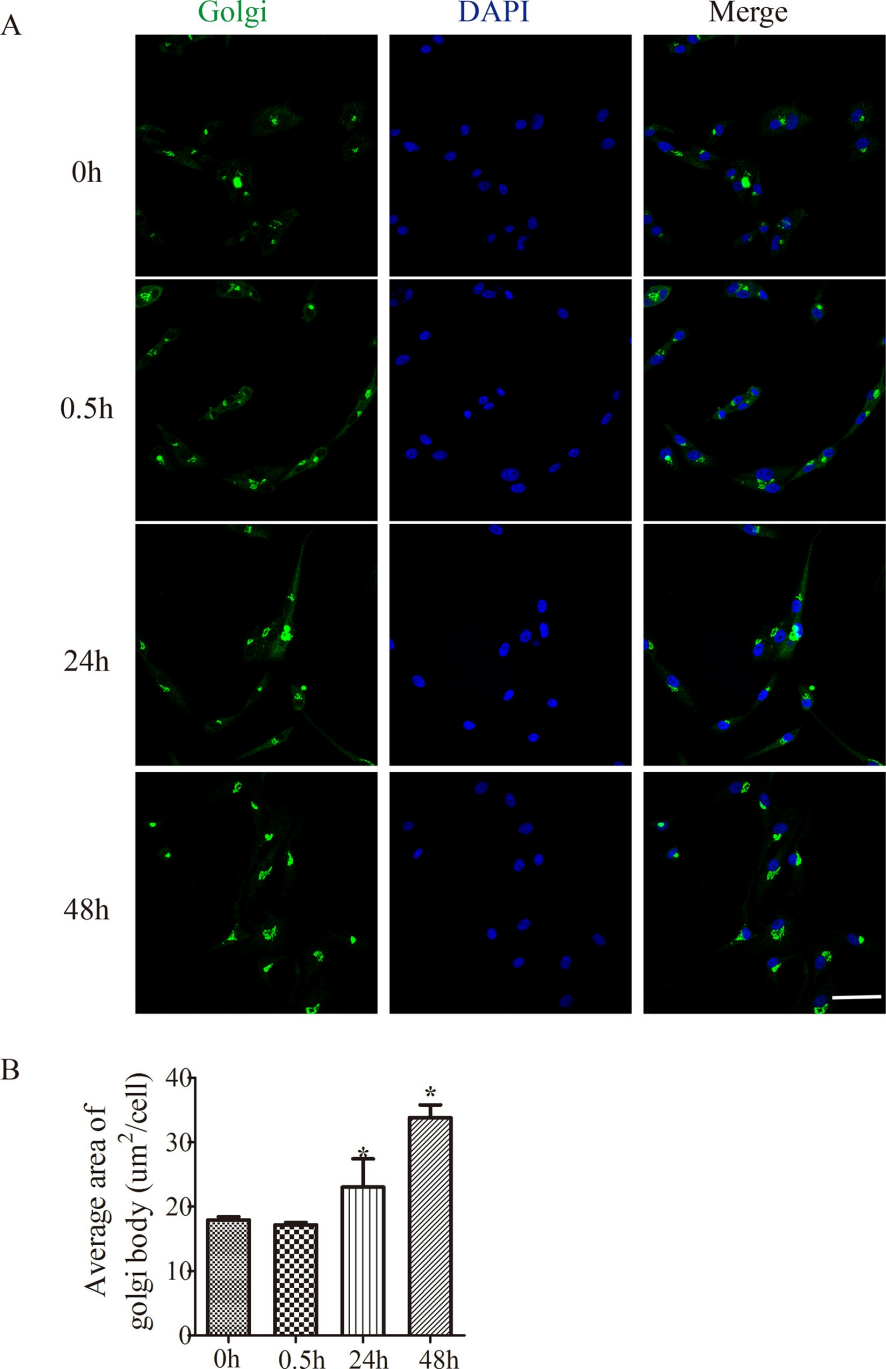
The stack area of the GA was increased over time fllowing GDNF treatment. (A) U251 cells treated with 50 ng/ml GDNF for 0.5, 24 and 48 h and the control group were stained and imaged to detect the GA. Golgi-ID green staining showing the GA (green), nucleus (blue) and merged images (bar = 50 μm). (B) Quantitative analysis of the area of the GA is presented as the mean ± standard deviation of three independent experiments. Compared with the control group, the average area of the GA body was increased by GDNF treatment at 24 and 48 h (*P<0.05). GA, Golgi apparatus; GDNF, glial cell line-derived neurotrophic factor.

To explore the effects of GDNF on Golgi apparatus, Golgi-ID green staining was performed to determine the changes of the GA in U251 cells treated with 50ng/ml GDNF for 0, 0.5h, 24h or 48h. The results showed that fragmented GA was reduced, and the GA concentrated and gathered near the nucleus in U251 cells following GDNF treatment for different time points (Fig 1A). Compared to non-GDNF-treated U251 cells (0h), the average stack area of the GA increased significantly and was more concentrated around the nucleus in U251 cells treated by GDNF for 24 and 48h (Fig 1B, *P<0.05).

### 2. GDNF increased the expression of golgin-160 and GMAP-210 in U251 cells

**Fig 2:**
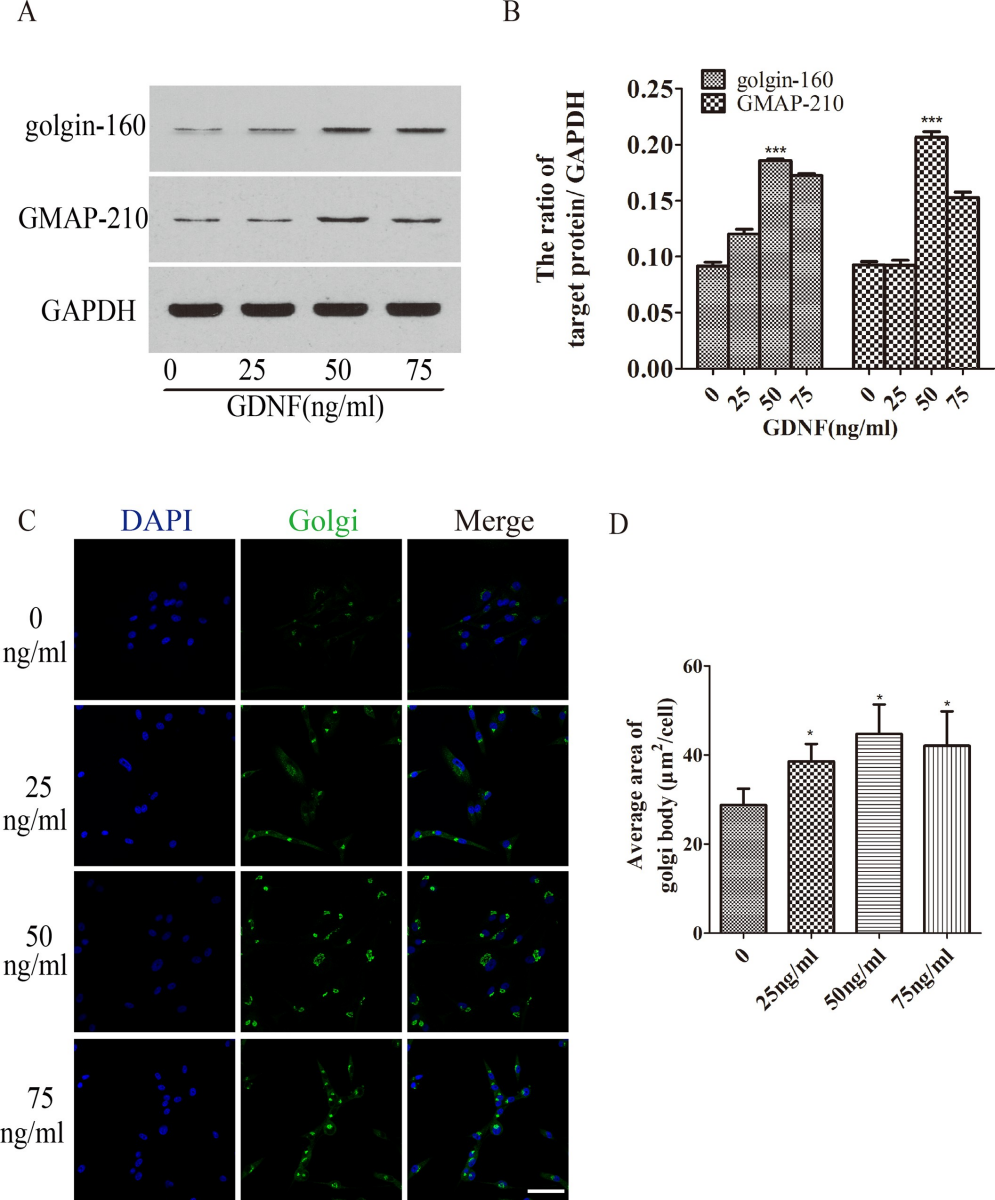
Increased expression of golgin-160 and GMAP210 and increased stack area of the GA were both induced by GDNF in a dose- dependence manner. (A and B) Western blotting detected the expression of golgin-160 and GMAP-210 in U251 cells treated by 0, 25, 50 and 75 ng/ml GDNF for 48 h. The expression of golgin-160 and GMAP-210 was increased most significantly following treatment 50 ng/ml GDNF (***P<0.001). (C and D) Immunofluorescence analyzed the stack area of the GA in U251 cells treated with 0, 25, 50 and 75ng/ml GDNF for 48 h. The GA area in U251 cells was increased following GDNF treatment, and it was significant following treatment with 50 ng/ml (*P<0.05; bar = 50 μm). GMAP-210, Golgi microtubule-associated protein 210; GA, Golgi apparatus; GDNF, glial cell line-derived neurotrophic factor.

To confirm the results above and further determine the best concentration of GDNF, western blot was used to detect the expression of golgin-160 and GMAP-210 in U251 cells treated by different concentration of GDNF (0, 25, 50, 75ng/ml) for 48h. The results showed that protein expression of golgin-160 and GMAP-210 were elevated in the GNDF treatment groups (Fig 2A), which was consistent with the increase in the stack area of the GA by Golgi-ID green staining (Fig 2C). The expression of golgin-160 and GMAP-210 were the highest in U251 cells treated with 50ng/ml GDNF (Fig 2B, ***P<0.001), and the stack area of the GA achieved the highest level when cells were treated with 50 ng/ml GDNF (Fig 2D, *P<0.05). No statistical difference was observed among the 25, 50 and 75ng/ml treatment groups.

### 3. GDNF promoted U251 cell migration and invasion influenced by depletion of golgin-160 or GMAP210

**Fig 3:**
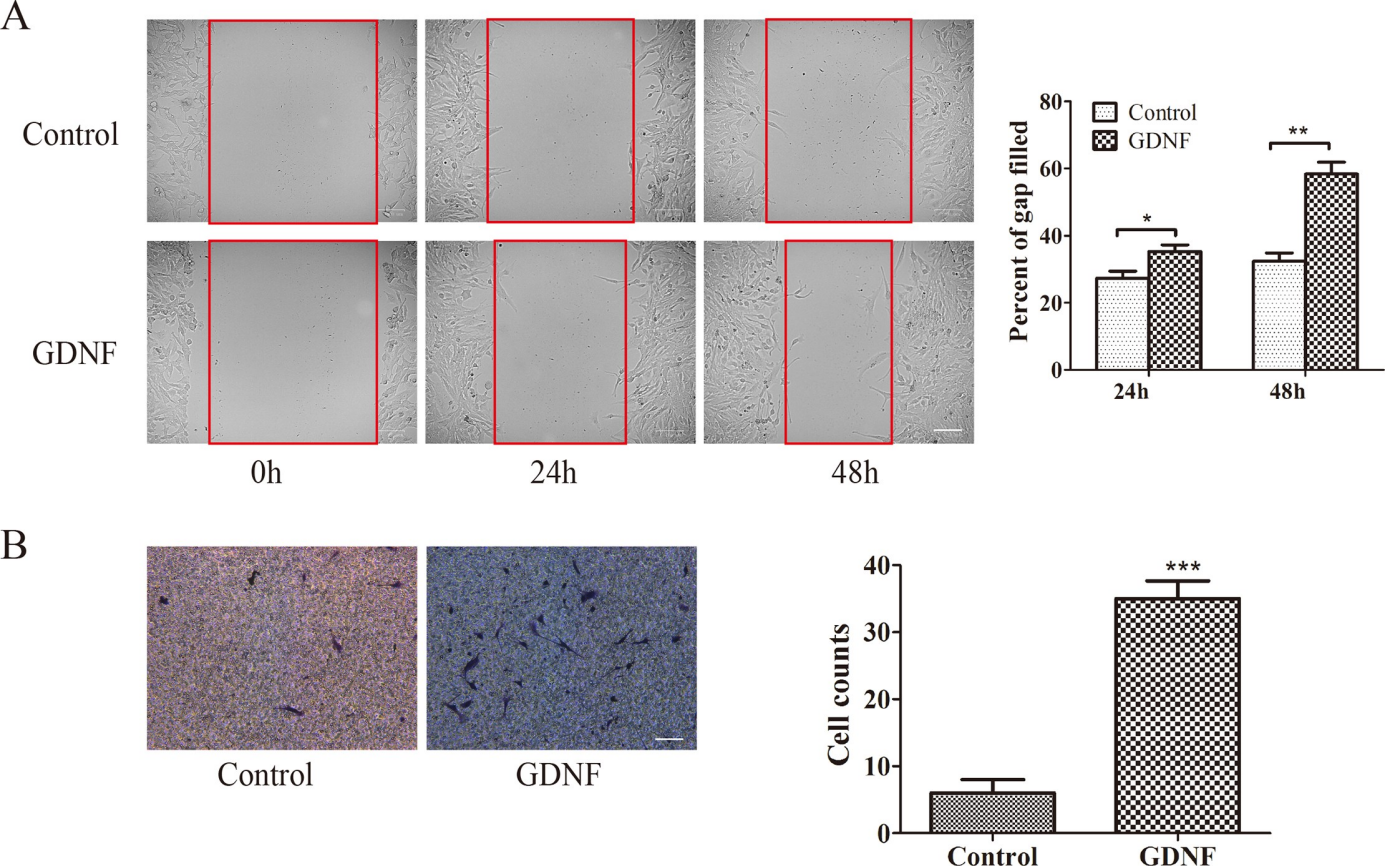
U251 cell migration was promoted by GDNF. (A) The effect of GDNF on U251 cell migration was assessed by wound healing assay (*P<0.05, **P<0.01, bar = 100μm). (B) The effect of GDNF on U251 cell invasion was assessed by Transwell invasion assay (***P<0.001; bar = 100 μm). GDNF, glial cell line-derived neurotrophic factor.

Based on our previous study [9] and the determination of the optimal treatment time and dosage of GDNF, we speculated that GDNF-facilitated U251 cell migration would be regulated by golgin-160 and GMAP-210. To test this speculation, wound healing and transwell invasion assay were conducted. The results showed that the gap-filling rate in the GDNF treatment group was higher than that in the control group, following scraping for 24h (Fig 3A, *P<0.05). Furthermore, the difference in the percentage of gap-filled was more significant at 48h (Fig 3A, **P<0.01). The Transwell invasion assay results showed that the number of penetrated cells after treatment with 50ng/ml GDNF for 24h increased, as compared with control group (Fig 3C), which was consistent with the results of our previous study [9]. To improve the persuasion of results, whether cell proliferation interferes with migration was explored additionally. As shown in S1 Fig, when cells were treated with non-serum, the proliferation was decreased significantly compared with serum group. Indeed, cell proliferation was improved only when GDNF was added (*P<0.05). Rather, once DNA inhibitor was used, even adding GDNF could not increase proliferation. As a whole, the effect of GDNF on cell proliferation was almost negligible through numerical analysis. Meanwhile, DNA inhibitor inhibited proliferation ultimately, which was little difference in proliferation from serum-free group. All the above detailed data were shown in the S1 Table. More plausibly, short-time time-lapse live imaging of cell motility was performed (S2 Fig), which was used to identify that GDNF could enhance cell motility in a spatially controlled manner absolutely. The results suggested that GDNF promoted cell motility obviously, which was consistent with S1 Fig (*P<0.05). Video data was separately uploaded as supplementary result (S1 Video). From the video, cell motility was significantly increased by GDNF; the wound healing was quicker than control group. Based on these, we made a brief summary: The ability of GDNF promoting cell migration could not be masked by cell proliferation.

**Fig 4:**
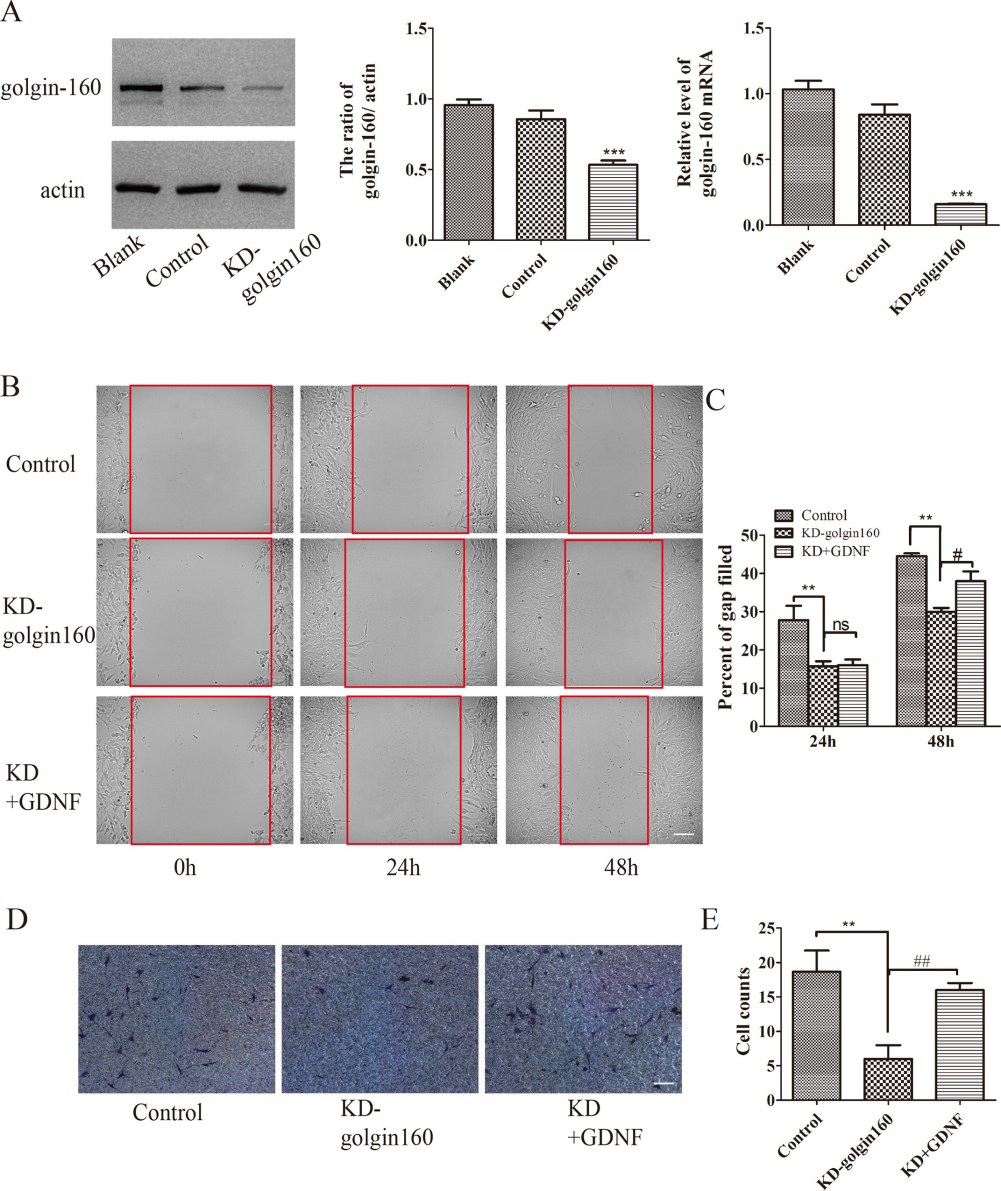
Effects of golgin-160 knockdown on U251 cell migration and invasion. (A) Golgin-160 was knocked down by lentivirus, and its expression was assessed by western blotting and RT-qPCR (***P<0.001). The effect of golgin-160 knockdown on U251 cell (B and C) migration and (D and E) invasion was assessed by wounding healing and cell invasion assays. Cell migration and invasion were inhibited by the depletion of golgin-160, and GDNF could improve it (##P<0.05 and **P<0.01; bar = 100 μm). RT-qPCR, reverse transcription quantitative polymerase chain reaction; GDNF, glial cell line-derived neurotrophic factor.

**Fig 5:**
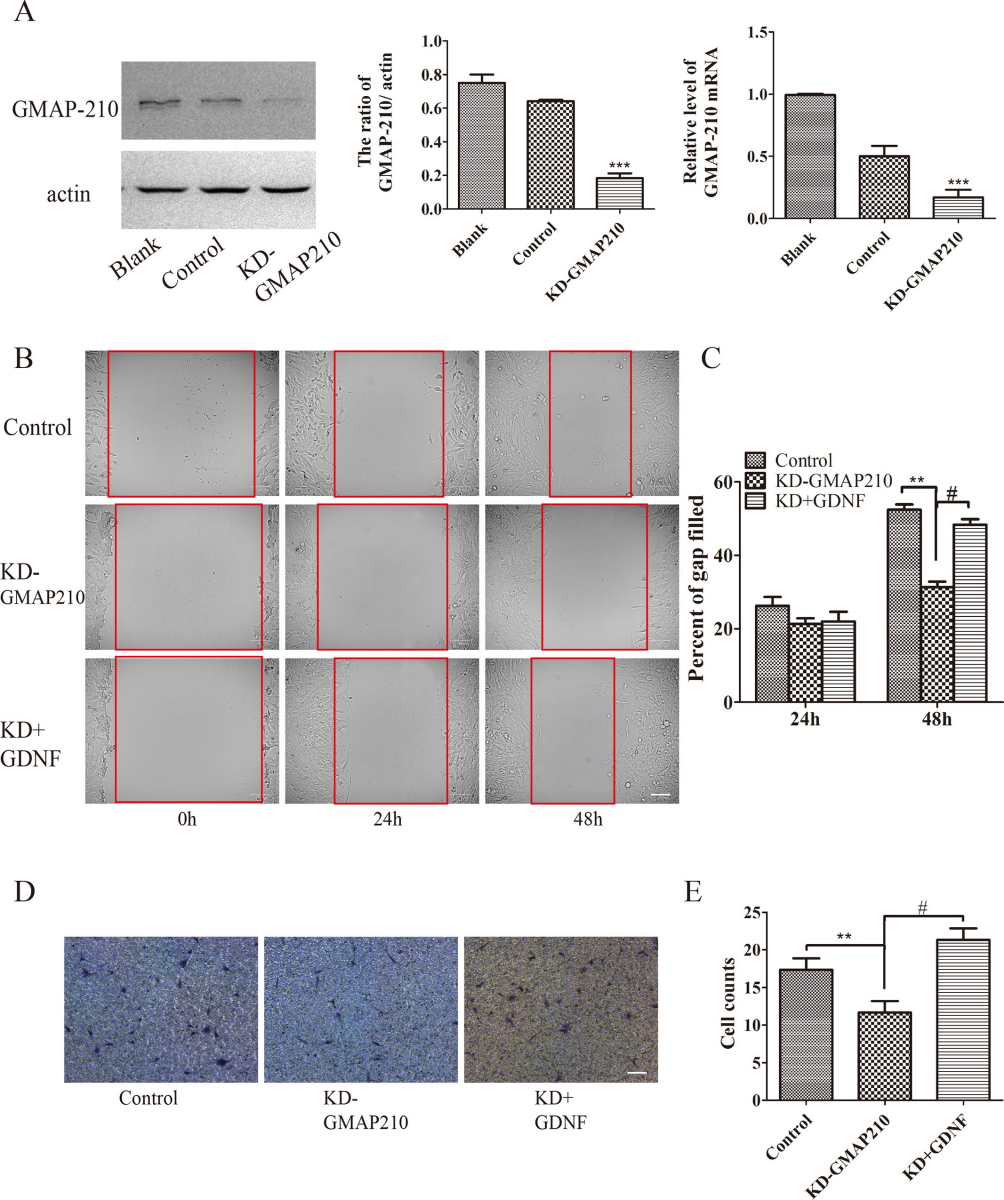
The depletion of GMAP-210 inhibited U251 cell migration and invasion, while GDNF improve it. (A) GMAP-210 was knocked down by lentivirus and its expression was assessed by western blotting and RT-qPCR (***P<0.001). The effect of GMAP-210 knockdown on U251 cell (B and C) migration and (D and E) invasion was assessed by wounding healing and cell invasion assays. Cell migration and invasion were inhibited by the depletion of GMAP-210, and GDNF could improve it (#P<0.05 and **P<0.01; bar = 100 μm). GMAP-210, Golgi microtubule-associated protein 210; GDNF, glial cell line-derived neurotrophic factor; RT-qPCR, reverse transcription quantitative polymerase chain reaction.

To determine whether the golgin-160 or GMAP210 contributes to the effect of GDNF on migration and invasion of U251 cells, we constructed lentivirus and stable cell line was screened by the puromycin. The western blot and RT-PCR experiments showed that knockdown of golgin-160 (Fig 4A) or GMAP210 (Fig 5A) had been successful. Simultaneously, cell migration was evaluated by scratch wound assay. The results demonstrated that down-regulation of golgin-160 or GMAP210 could decrease cell migration (Figs 4B, 4C, 5B and 5C **P<0.01,). When stable line cells resumed treating with GDNF, their migration ability was improved, as compared with that of cells in the knockdown golgin-160 (or GMAP210) group. However, their ability was still worse than that of cells in the control group at 48 h (#P<0.05), while there was no difference between these two groups at 24h (Figs 4C and 5C). Subsequently, cell invasion was explored *in vitro*. Similarly, regardless of the protein that was knocked down, cell invasion was impaired (Figs 4D and 5D; **P<0.01,). In addition, the number of penetrated cells was increased with the action of GDNF (Figs 4E and 5E; #P<0.05).

### 4. Depletion of GMAP210 yields more fragments in the GA and drives it away from the nucleus

**Fig 6:**
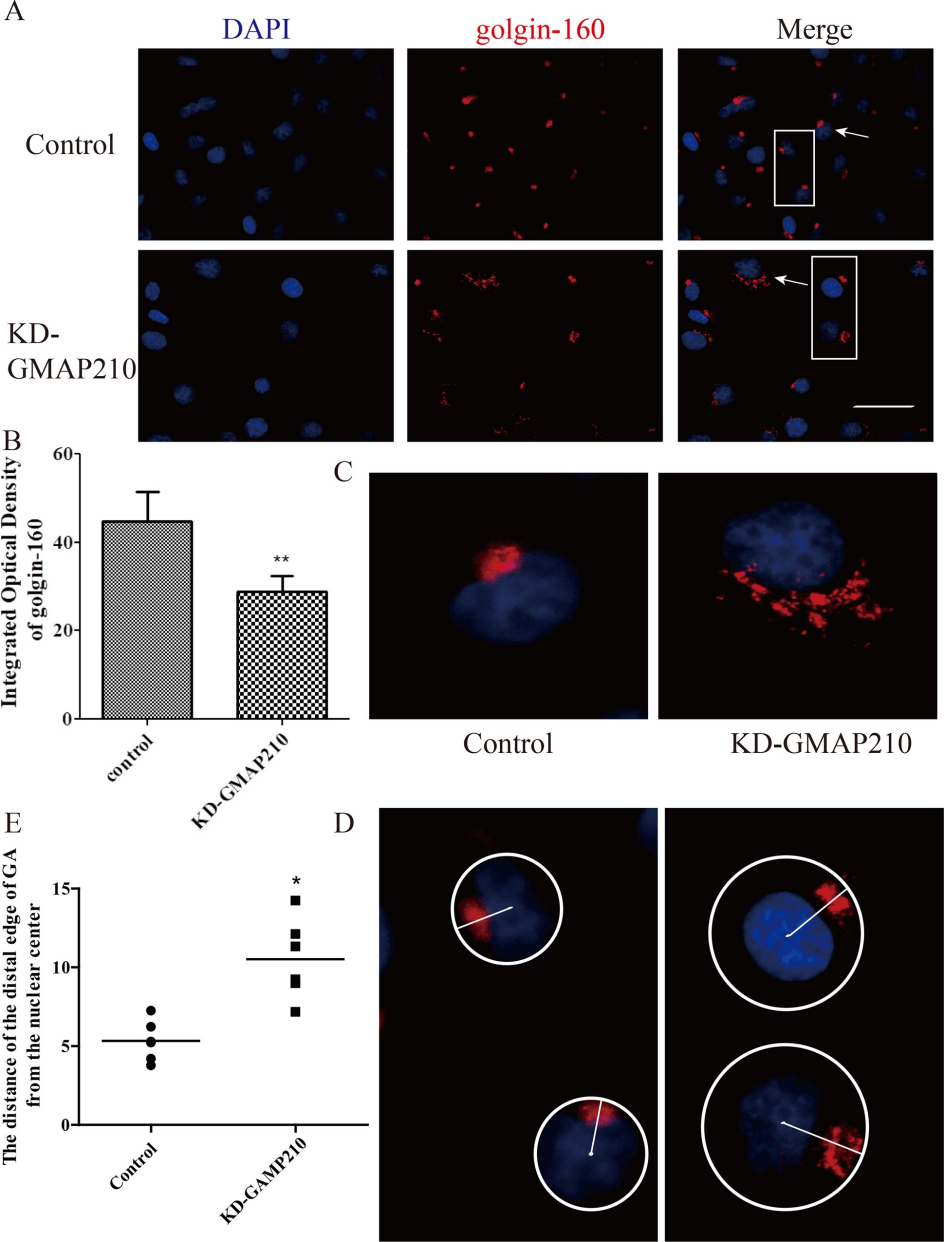
Morphological observation of the GA and distance detection from cell nucleus through Immunofluorescence. (A and B) Immunofluorescent images and analysis of golgin-160, which was considered a Golgi marker protein. Red (golgin-160), blue (nuclear) and merged images (bar = 50 μm). Following golgin-160 knockdown, the integrated optical density of golgin-160 was decreased, while the GA profile was still visible (**P<0.01). (C) Zoom in to the specific cells from A (arrow pointing); it was shown that the increased dispersion level of the GA was due to the depletion of golgin-160. (D and E) Zoom in to the specific cells from A (rectangle); the distance from the remote margin of the GA to the cell nucleus was detected using Image Pro Plus. Each dot represents the distance from the GA to the nucleus of different cells. The different values were plotted (μm); the KD-Golgi-160 group was father from the nucleus (*P<0.05). GA, Golgi apparatus.

Based on the confirmation that cell migration and invasion were inhibited by the depletion of golgin-160 or GMAP210, changes in the GA, which is one of the most important organelles in regulating cell migration and invasion, were observed. Immunofluorescence was performed to localize golgin-160 protein, a member of the Golgi family localized to the GA. The results showed that the fluorescence intensity of golgin-160 was clearly decreased (Fig 6A and 6B), and the difference was statistically significant (**P<0.01). In addition, as compared with control group, the distinct fluorescent Golgi mini-objects were increased by the depletion of GMAP210. Fig 6C shows the dispersed Golgi membranes and fragments. Next, the distance from GA to the nucleus was measured. It was found that golgin-160 or GMAP210 knockdown blocked the peri-centrosomal positioning of the GA[16]. The results showed that the distance from the distal edge of GA to the nuclear center was increased, due to the decreased expression of GMAP210 (Fig 6D and 6E, *P<0.05). Knockdown cells depleted by either golgin-160 or GMAP210 behaved in a similar manner, which was verified by the results presented in the next section.

### 5. GA Agglutination level was affected by golgin-160 and the agglutination level was improved in U251 cells following GDNF treatment

**Fig 7:**
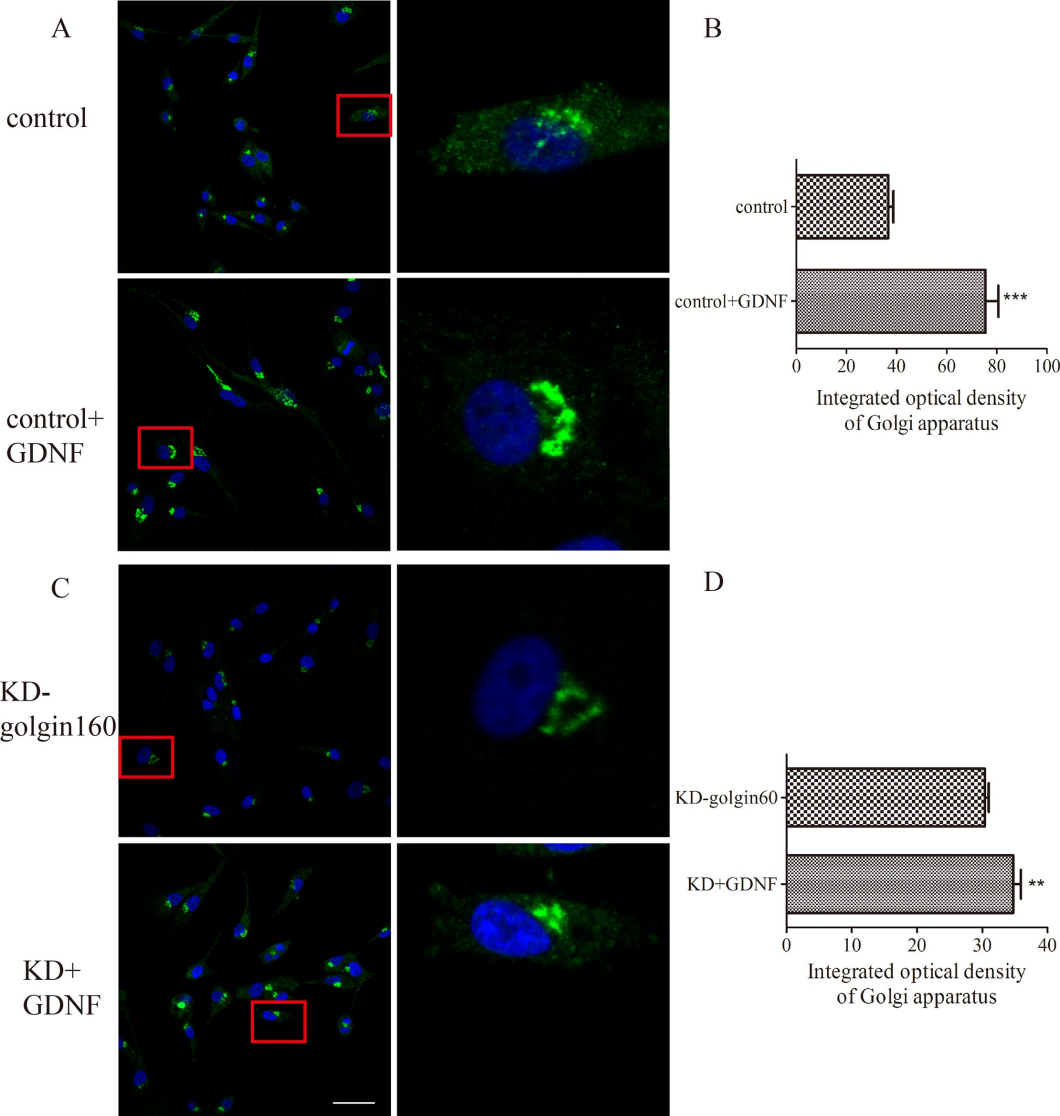
Golgi-160 was essential for agglutination level of the GA and GDNF improved it. (A and B) Immunofluorescence and GA agglutination level analysis in the control group, with or without 50 ng/ml GDNF treatment. Following GDNF intervention, the agglutination level of the GA was more prominent (***P<0.001; bar = 100 μm). (C and D) Immunofluorescent images and GA agglutination level analysis in the KD-golgin160 group, with or without 50 ng/ml GDNF treatment. The integrated optical density of the GA was decreased and its morphology was diffused due to the depletion of golgin-160. GDNF improved the agglutination level (**P<0.01). For better observation and analysis, we zoomed in to the images of selected target cells (red rectangle). Bar = 50μm. GA, Golgi apparatus; GDNF, glial cell line-derived neurotrophic factor.

To confirm the complete morphology of Golgi, the effect of exogenous GDNF on the GA when golgin-160 was knocked down in U251 cells was also explored through Golgi-ID green staining. The results proved consistent with those of glogin-160 staining above. It was verified that GDNF could promote U251 cell migration and invasion, which required the mediation of golgin-160 and GMAP210. Since the depletion of golgin-160 generated GA fragmentation and inhibited migration and invasion, the change in the GA in cells treated with 50ng/ml GDNF for 48h was observed. Consistent with our speculation, the agglutination level of the GA was increased by GDNF stimulation (Fig 7A and 7B), which was reflected by the analysis of integrated optical density (IOD) of the GA (***P<0.001). Consistent with the previous results (Fig 6), the depletion of golgin-160 actuated the GA dispersion but rather may abate agglutination level of the GA, as compared with the control group (Fig 7A–7C). When GDNF was replenished based on the knockdown of golgin-160, the agglutination level of the GA was improved significantly (Fig 7C and 7D), which was reflected by the findings of the GA IOD analysis (**P<0.01). Altogether, the results of the present study demonstrated that GDNF promotes U251 cell migration and invasion through the mediation of the GA orientation and agglutination by golgin-160 and GMAP-210.

## Discussion

In the present study, it was found that the incubation of cells with GDNF leads to an increased cell migration and invasion capability. As a result, the GA counts much in regulating directional cell migration mediated by golgin-160 and GMAP210, which organize Golgi mini-stacks. Cell migration is a dynamic and highly complicated cellular process. Deregulation of migration is the main characteristic of cancer, and glioma is no exception.

Studies have shown that Golgi orientation is important for cell polarization and migration, as a polarized Golgi supplies membrane components for leading edge protrusion or material for apical secretion[18]. Based on this study and our previous study [9], we speculated that GDNF could raise the agglutination level of GA and lead it towards the nucleus. This hypothesis was confirmed by the present results.

In mammalian cells, the GA is polarized in both structure and function and it is localized to juxta nuclear zone[19]. The Golgi itself participates in a variety of protein modification processes as well as the supply of components to the cell membrane. During migration, the changes of cell membrane polarization accompanied with the continuous and dynamic changes of the component in the front membrane. Therefore, it is worth determining the GA position and the direction of cells movement during cell migration. When GDNF was added to the U251 cells, they secreted more MMP9 [9], which degrades ECM and basement membrane components and promotes their invasion and metastasis [20]. Both the results of a previous and this study showed that cell migration was likely influenced by the polarity of the position and function of the GA following GDNF treatment, which led to GA reorientation toward the migration leading edge and secretory trafficking [14]. In addition, GDNF promotes MMP-9 secretion in U251 cells [9]. We speculated that the secretion of MMP9 was also in the direction of leading edge, which facilitates cell infiltration. However, this mechanism requires further exploration.

In the present study, the entire structure of the GA was observed; it appeared to be in a diffuse state and to have undergone significant changes following the knockdown of golgin-160. The GA was also found further away from the nucleus. These observations were consistent with those of the previous study[16]. Numerous studies have confirmed that GA is one of the most important organelles involved in directional cell migration[21]. It is also associated with asymmetric cytoskeletal arrangement[22], intracellular organelle localization, membrane domain segregation[23] and polarized cell morphology[24, 25]. What could regulate the Golgi reorientation? A matter of sparking and intense research was executed. Primarily, disassembling the GA leads to its failure to orient towards the leading edge, which further damages the centrosome reorientation [26]. Hurtado *et*.*al* reported that both the centrosome and the GA failed to reorient towards the leading edge, and directional migration was strongly inhibited by knocking down AKAP450[27]. That is the cause of the destruction of the polarity of the GA and the centrosome. The centrosome is also known as an microtubule-organizing center (MTOC)[28], which determines the positioning of GA [29]through MTs such as tubulin[19]. A different study also identified that the AKAP450, which is involved in the targeted positioning, is critical for cellular functioning, particularly in MT nucleation and cell migration[30].

Astrocytes change from sparsely branched cells to polarized and highly ramified cells [31] during development, which requires the polarity of the centrosome and GA. In addition GDNF plays an important role in astrocyte development. In our study, it was observed that GDNF could promote glioma cell migration and increase the expression of GA-related proteins. Following the knockdown of golgin-160 or GMAP210, the polarity orientation of GA was destroyed and cell migration was inhibited. Even so, what do the golgin-160 and GMAP210 work on earth in cell migration and whether it has a relationship with cell polarity of GA or not? Tubulin endows the dynamics and polarity of Golgi apparatus and cell itself, which makes it produce migration power. Studies have shown that once the microtubules depolymerize, the Golgi apparatus is not a ribbon vesicle, but is broken up into small heaps[32].

Golgin-160 and GMAP210 are proteins with key roles in GA positioning and function[16]. The GA has an assembly center of α/β-tubulin acting as MTOC[33, 34]. Of note, the depletion of GMAP210 or golgin-160 leads to the failure to recruit γ-tubulin, which causes further destruction around the centrosome, GA fragmentation and inhibition of cell migration [16]. Another study has also indicated that golgin-160 ensures efficient delivery of protein-processing to the surface from the trans-Golgi network [35]. GMAP210 acts at the endoplasmic reticulum (ER)-to-Golgi during anterograde trafficking, and is also required for retrograde trafficking to the ER[36]. That demonstrates the importance of golgin-160 and GMAP210 in the GA peri-centrosomal positioning and cell migration. GDNF increased the expression of golgin-160 and GMAP210 in U251 cells. However, the specific mechanism of GDNF on the GA is still unknown. In summary, establishing conditions to test the specific role of GA positioning on directed secretion and glioma cell polarity following GDNF treatment should be further investigated. It should be further explored that GDNF regulates GA reorientation and how it affects cell polarity.

## Conclusion

The regulation of the positioning and size of the GA by golgin-160 and GMAP210 plays an important role in GDNF promoting U251 cell migration and invasion.

## Supporting information

S1 Fig Relative proliferation and migration of U251 cells treated with GDNF, DNA inhibitor (mitomycin C). (A) Cell proliferation was assessed by CCK8. In serum-free medium, GDNF increased the proliferation moderately (*P<0.05) compared with other Intervention conditions. There was no difference in cell proliferation between DNA inhibitor group and serum-free + DNA inhibitor group.(B and C) Wound healing assay was used for comparison of cell migration. Both serum-free +GDNF and Serum-free +DNA inhibitor +GDNF groups performed greater mobility in cell migration than other two groups (***P<0.001). There was no difference between serum-free +GDNF and Serum-free +DNA inhibitor +GDNF groups.
(TIF)

S2 Fig Live cell imaging of cell motility. At 6^th^ h after scratching, start recording through Olympus IX81 inverted microscope with a new UIS2 optical system. The duration of recording was from 6^th^ to 48^th^ h. 0s represents the starting point of recording (The actual time is 6^th^ h after the scratching); 12s represents the end point of recording (The actual time is 48^th^ h after the scratching).
(TIF)

S1 Video Video data of cell motility in control and GDNF groups. (ZIP)

S1 Table The OD_450_ data comparison among different groups (mean±SD). (DOCX)
