# Supplementary figures and images for: Golgin-160 and GMAP210 play an important role in U251 cells migration and invasion initiated by GDNF
Source: PLoS One. 2019 Jan 29;14(1):e0211501. doi: 10.1371/journal.pone.0211501 (PMC6351060; doi:10.1371/journal.pone.0211501)

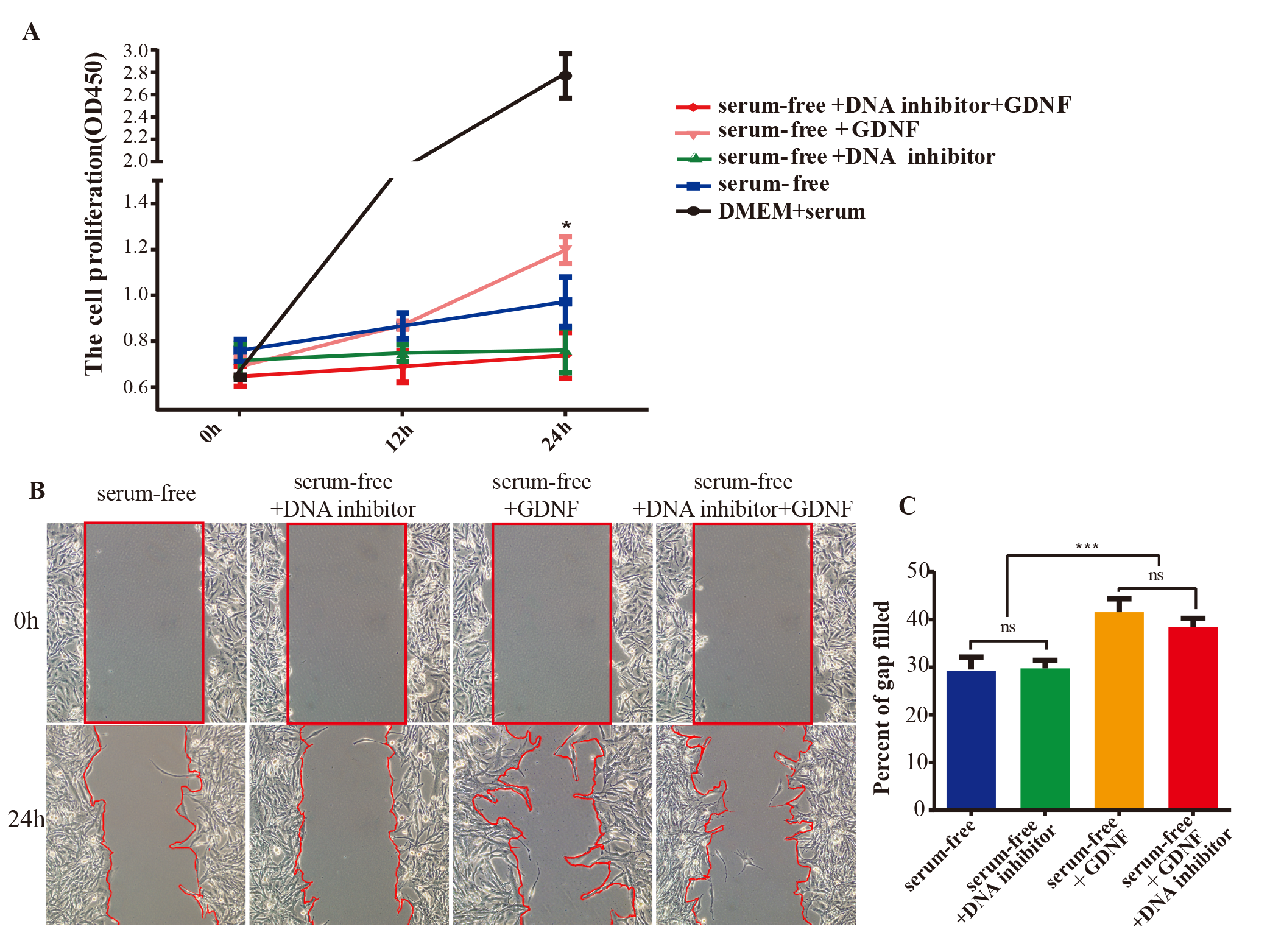

Supplement: S1 Fig — (A) Cell proliferation was assessed by CCK8. In serum-free medium, GDNF increased the proliferation moderately (*P<0.05) compared with other Intervention conditions. There was no difference in cell proliferation between DNA inhibitor group and serum-free + DNA inhibitor group.(B and C) Wound healing assay was used for comparison of cell migration. Both serum-free +GDNF and Serum-free +DNA inhibitor +GDNF groups performed greater mobility in cell migration than other two groups (***P<0.001). There was no difference between serum-free +GDNF and Serum-free +DNA inhibitor +GDNF groups. (TIF) [file pone.0211501.s001.tif]

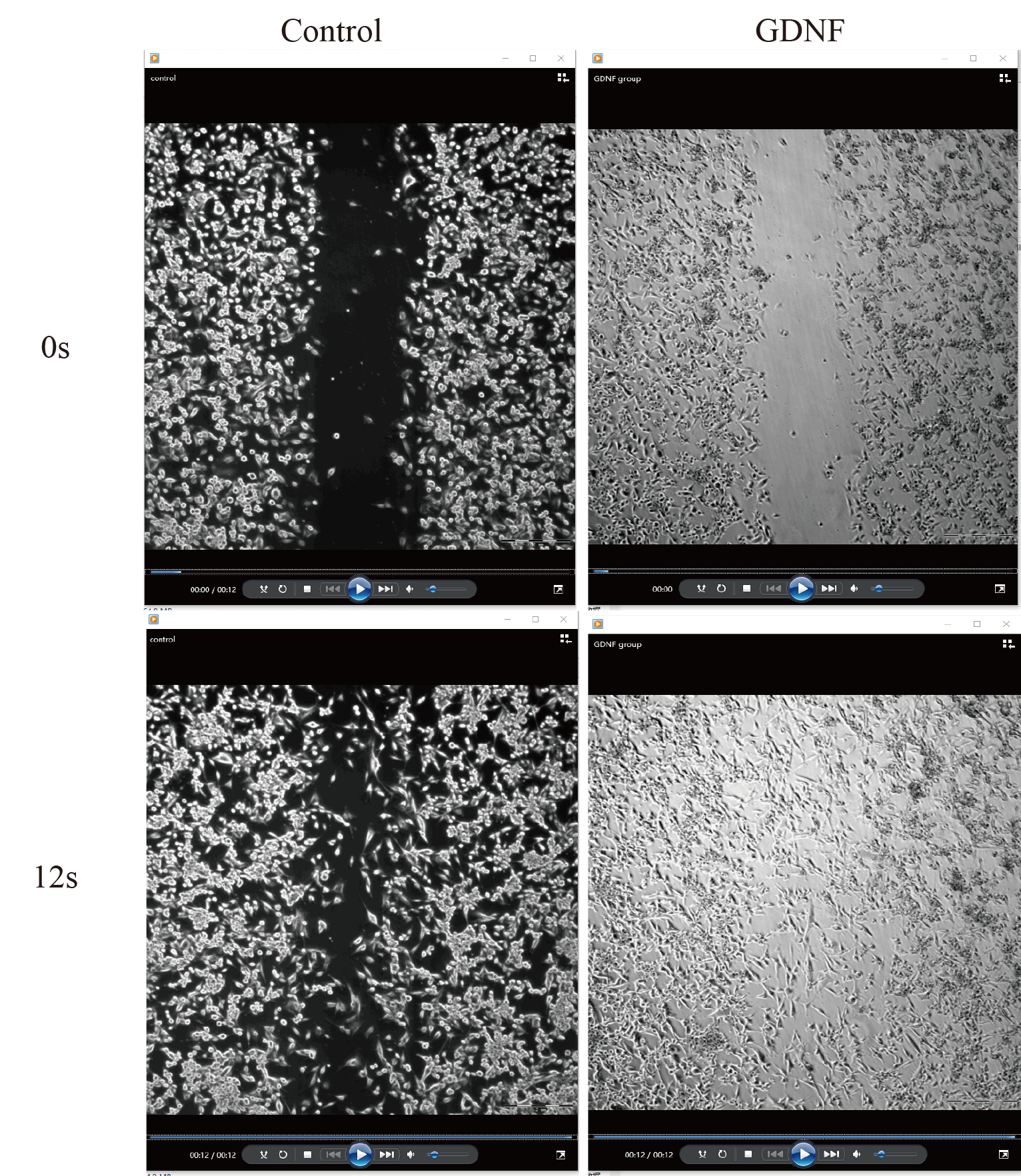

Supplement: S2 Fig — At 6th h after scratching, start recording through Olympus IX81 inverted microscope with a new UIS2 optical system. The duration of recording was from 6th to 48th h. 0s represents the starting point of recording (The actual time is 6th h after the scratching); 12s represents the end point of recording (The actual time is 48th h after the scratching). (TIF) [file pone.0211501.s002.tif]
