# Supplementary material for: Golgin-160 and GMAP210 play an important role in U251 cells migration and invasion initiated by GDNF
Source: PLoS One. 2019 Jan 29;14(1):e0211501. doi: 10.1371/journal.pone.0211501 (PMC6351060; doi:10.1371/journal.pone.0211501)
Supplement: S1 Table — (DOCX) [file pone.0211501.s004.docx]

Table S1. The OD_450_ difference comparison at different check points（Mean±SD）

| OD_450_ | 0h | 12h | 24h |
| --- | --- | --- | --- |
| DMEM+serum | 0.643±0.058 | 1.562±0.061 | 2.768±0.202 |
| serum-free | 0.759±0.048 | 0.867±0.057 | 0.972±0.108 |
| serum-free +DNA inhibitor | 0.717±0.073 | 0.748±0.366 | 0.760±0.978 |
| serum-free +GDNF | 0.689±0.039 | 0.870±0.174 | 1.197±0.058 |
| serum-free +DNA inhibitor  +GDNF | 0.648±0.043 | 0.689±0.069 | 0.737±0.104 |
